# Quantitative Measurement of Throat and Larynx After Endotracheal Intubation for Palatoplasty

**DOI:** 10.3389/fmed.2022.745755

**Published:** 2022-03-28

**Authors:** Pei-Rong Lee, Chung Feng Jeffrey Kuo, Shao-Cheng Liu

**Affiliations:** ^1^Department of Otolaryngology-Head and Neck Surgery Tri-Service General Hospital, National Defense Medical Center, Taipei, Taiwan; ^2^Department of Materials Science and Engineering, National Taiwan University of Science and Technology, Taipei, Taiwan

**Keywords:** quantitative laryngoscopy, computer-aided diagnostic system, tracheal intubation, palatoplasty, oropharyngeal inlet

## Abstract

**Introduction:**

Quantitative morphometric measurements of living human upper airway remain challenging. This study aimed to introduce a special laser projection device that can facilitate computer-assisted, digitalized analysis and provide important information on airway mucosa change, before and after endotracheal intubation for palatoplasty.

**Materials and Methods:**

The laryngeal images were captured before and after surgery. Image processing techniques were used to quantize the post-operative laryngeal variation, with its distinct color space and texture features. Meanwhile, the maximum length of the vocal fold, vocal width at the midpoint, maximum cross-sectional area of the glottic space, maximum cross-sectional area of the oropharyngeal inlet (CSAOI) and the depth of the retropalatal space were determined and calculated. These parameters were analyzed and compared before and after surgery.

**Results:**

A total of 39 subjects were enrolled in this study. The color space and texture analysis all show trends toward higher measures in post-operative images than in pre-operative images, especially in the interarytenoid region. Furthermore, the glottic area showed a significant decrease of 31.2%, while the vocal width showed a significant increase after intubation. The post-operative retropalatal depth and CSAOI were significantly deeper and larger than the baseline, reaching their peak in the 4th week after the surgery, and then slightly reduced in the 12th week.

**Conclusion:**

For the first time we present a series of changes in upper airway after surgery, by using a laser module with quantitative measurement. Our equipment and processing can measure subtle mucosal changes that would allow a clinician to diagnose post-operative airway inflammation in a simpler and less invasive way. Here additional information collected by different imaging modalities would help to solve multiple current unmet needs in post-operative airway inflammation.

## Introduction

Laryngeal adverse reactions are a well-recognized consequence of endotracheal general anesthesia (1). A broad range of symptoms can be encountered and post-operative hoarseness, an clinical sign of laryngeal injury or dysfunction, can be distressing to a patient (2). In severe cases, it may be associated with serious consequences such as difficulty in swallowing and respiratory distress (3). The incidence of these post-extubation sequelae may be underestimated, as patients usually consult an otolaryngologist only when hoarseness persists for a prolonged period. Laryngoscopy is the most commonly used diagnostic method, but it is inaccurate for minor changes and the interpretation is subjective. To date, there is still no consensus in the literature on how to objectively measure changes in the larynx after endotracheal general anesthesia.

An alternative to detect laryngeal pathologies would be to observe and record changes in metric dimensions or physical appearance, such as mucosal color, and texture (4). Due to lack of reliable scaling conversion references to learn the corresponding relationship between image pixels and length units, it was very difficult to measure the absolute value of the human upper aerodigestive tract by endoscopic images. Over the past decades, few studies have been conducted to elucidate the association between mucosa appearance and inflammation. Accordingly, we are committed to finding a real-time and low invasive alternative, that can be implemented immediately in an outpatient setting. We have once introduced a new device composed of a rigid laryngoscope and laser pointers, which was the first report to measure the absolute values of the laryngeal and oropharyngeal structures (5, 6). Although there is evidence that this type of outpatient screening is quite convenient and provides objective quantitative information, the applicability of the device in patients after surgery with endotracheal general anesthesia is still unclear.

In past studies, patients who underwent palatoplasty were evaluated by Muller maneuver, Friedman stage, etc. Only semi-quantitative information could be provided. With our previous background knowledge, this study was conducted based on actual clinical data with absolute laryngeal and oropharyngeal measurement, which can be used in the analysis of mucosa change after palatoplasty under endotracheal general anesthesia. Hence, the primary aim of this study was to confirm whether our device has distinguishable measurements in patients before and after surgery. The secondary aim was to investigate the change of human upper aerodigestive tract after intubation and explore the association between inflammation and anatomic features, to determine whether laryngeal features could be potential predictors for post-operative inflammation. With more objective and convenient examinations, surgeons may tailor surgical techniques to cope with different clinical conditions, and can more accurately assess the impact of surgery on patients.

## Materials and Methods

### Equipment and Human Data Acquisition

**Figure 1:**
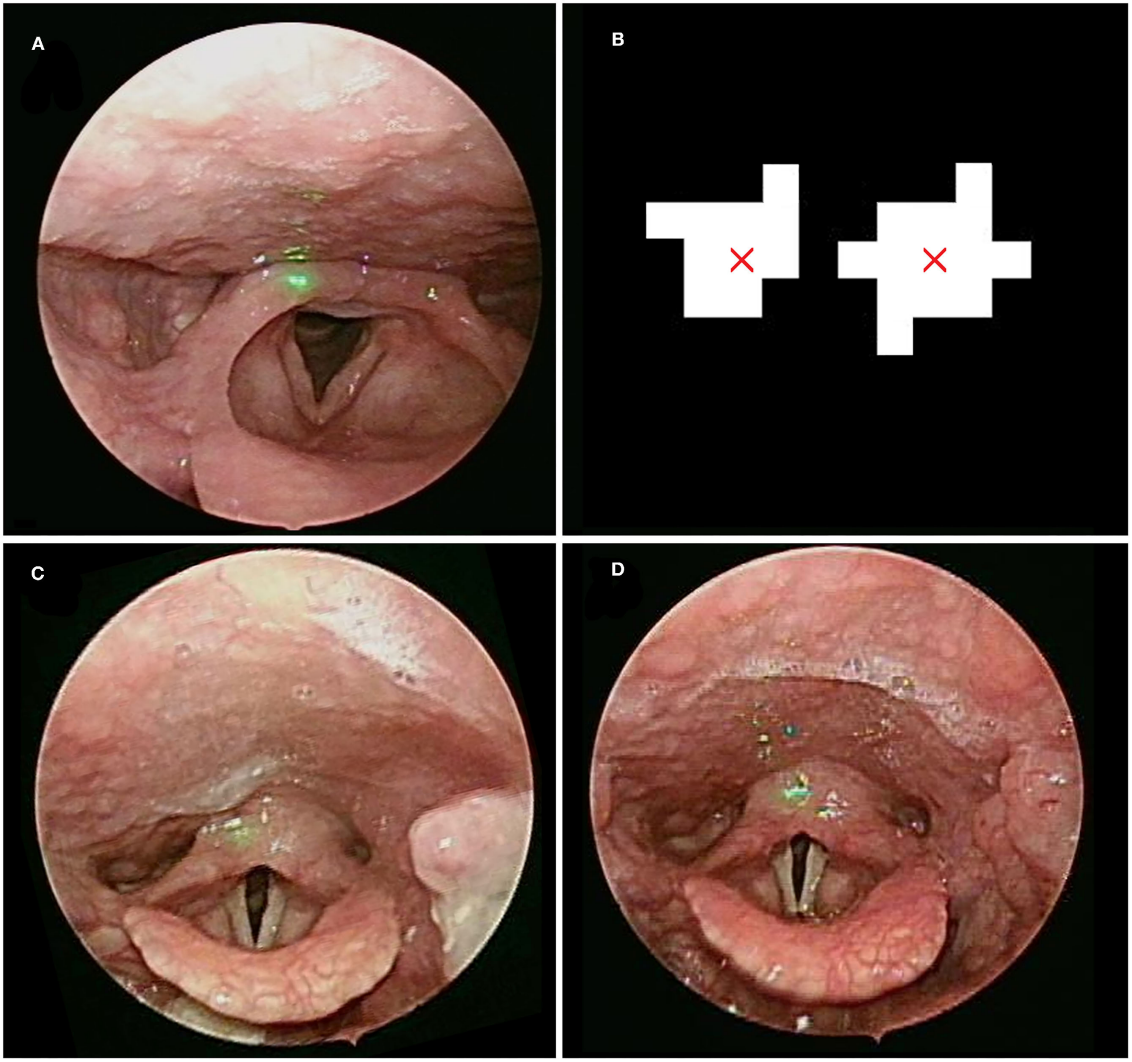
**(A)** The laser beams projected on the healthy control subject. **(B)** Image processing defines the center of the laser dot and gives us its equivalent distance as 1 mm. The laryngeal images before **(C)** and after **(D)** general anesthesia with intubation.

**Figure 2:**
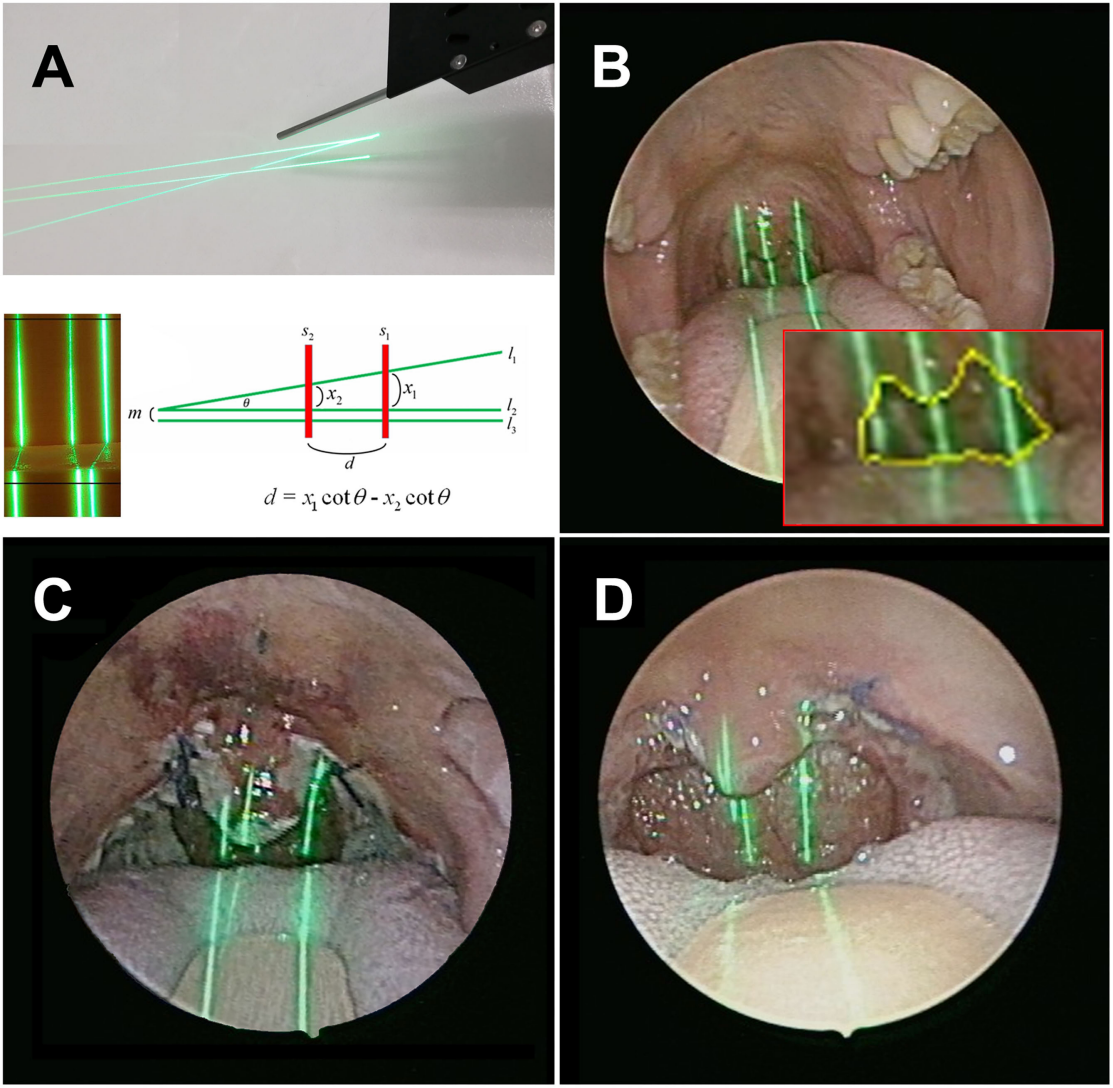
**(A)** A total of three line laser beams, including one at an angle of 8.5 degrees with the other two which were parallel, **(B)** The laser beams projected on the oropharynx pre-operatively. The retropalatal depth and the cross-sectional area were calculated automatically. Post-operative evaluation – week 1 **(C)** and week 12 **(D)**.

As in our previous report (5, 6), our module comprised a rigid 70° endoscope, laser pointers, and a reflection lens. Two parallel laser beams, with a distance of 1 mm, were obtained from a laser excitation apparatus (H435151D/R, 515 nm). The two parallel laser beams can provide the scaling reference for further image analysis (Figure 1A). To measure the oropharyngeal structure, laryngoscopy was performed with a device which composed of a rigid straight endoscope, three laser pointers and a grating sheet (6). The dot laser was transformed to a liner laser by the grating sheet, and provided the scaling reference. A total of three line laser beams, including one at an angle of 8.5 degrees with the other two which were parallel, were used to facilitate the calculation of the target depth (Figure 2A).

This study was conducted from March 1, 2020 to February 28, 2021. We collected laryngoscopic images from patients who underwent palatoplasty under endotracheal general anesthesia. The exclusion criteria were as follows: (1) had undergone any upper airway surgery except nasal surgery; (2) presence of other sleep disorders, such as insomnia, parasomnia, restless leg syndrome (RLS), etc.; (3) fiber-optic endoscopy revealed apparent nasal or retro-lingual obstruction, which were not easily corrected by pterygomandibular suspension suture; (4) length of out-patient department follow-up after surgery <3 months. After bilateral tonsillectomy, The participants underwent palatoplasty with additional sutures using 3-0 Polysorb™ (Medtronic) near the pterygomandibular suspension to increase the traction force on the soft palate and lateral pharyngeal wall. Complete or partial anterior and posterior tonsillar pillars were approximated with absorbable sutures, depending on appropriate tension over the pharyngeal wounds. For laryngeal measurement, laryngoscopy was performed before and within 24 h after surgery (Figure 1). For oropharyngeal measurement, post-operative regular out-patient department follow-up for at least 3 months (week 1, week 2, week 4, and week 12) was required for all participants (Figure 2). A minimum of 3 evaluations was carried out so the observer could acquire and determine the maximum value of oropharyngeal and laryngeal parameters, including the depth of the retropalatal space, the maximum cross-sectional area of the oropharyngeal inlet (CSAOI), the vocal width, length, area, maximum cross-sectional area of the glottic space, and maximum vocal fold angle, as shown in our previous work (5).

### Image Processing

#### Hue Feature and CIELab

The videos (1,920 × 1,080) were captured and processed by MATLAB. An endoscopic image is directly applicable to the RGB (red, green, blue) color space. Then, spectroradiometric objective color measurements and the CIELab is used for the variable of mucosal complexion as it is a color space modeled on the visual pattern of humans and designed to be perceptually uniform in human perceptual work (7). Coordinates of CIELab are L, a, and b, respectively, where L^*^ represents the lightness of the color (L^*^ = 0 indicates black and L^*^ = 100 indicates diffuse white), a^*^ represents the position between red and green (negative values means green and positive values means red) and b^*^ represents the position between yellow and blue (negative values means blue and positive values means yellow). Conversion between RGB and CIELab is a two stage process according to the equations of the previous study (8). The purpose of this CIELab color space is to enhance the color images better than RGB space, where it can represent an infinite number of ranges of chromatic than other color space models. Unlike most of the other objective color measuring methods, CIELab can provide highly accurate non-contact color readings, thus avoiding undesirable edge-loss errors characteristic of contact-type color measuring instruments.

#### Textual Features

In terms of the perceptual experience of the human eye, the rough and directionality are the primary characteristics used by the human eye to distinguish texture. The Gray-level Co-occurrence Matrix (GLCM) describes the grayness relationship between adjacent pixels in a local area or overall area of an image (9). To quantize the mucosal variation induced by inflammation, this study used the energy (equalization), contrast, correlation, and homogeneity of GLCM to describe the texture information of various regions of the larynx. The definition of our function index was calculated as follows:


$$\begin{array}{l} \text{Function index}\\ =\;\sqrt{0.35\text{CIELab}\hat{\;}\text{2}+\text{0.34}\text{Contrast}\hat{\;}\text{2}+\text{0.32}\text{Correlation}\hat{\;}\text{2}} \end{array}$$

### Statistical Analysis

Data were presented as the mean ± standard error of the mean (SEM). All statistical analyses were performed using Student's *t*-test or paired samples *t*-test on GraphPad Prism 5 software (GraphPad Software Inc., La Jolla, CA, USA); *P* <0.05 was considered statistically significant.

### Ethical Considerations

The research protocol (No: 1-108-05-132) was reviewed and approved by the Institutional Review Board of the Tri-Service General Hospital, Taipei, Taiwan. All methods were performed following the relevant guidelines and regulations. All patients provided written informed consents before participation.

## Results

Patients who could not continue with the post-operative follow-up, or whose images had a noticeable blur or glare, were omitted from this study. Finally, a total of 39 subjects were enrolled in this study. The ages of all participants were between 20 and 68 years old, and the mean age of these patients was 38.9 ± 6.1 years old, with no significant difference in age between both genders (37.6 ± 5.6 vs. 40.8 ± 25.3, *p* > 0.05). The tubes were all fixed in the midline area of the mouth during surgery. All patients were successfully extubated in the operation room, and no one experienced respiratory compromise. There was no major wound bleeding, wound dehiscence, nasal regurgitation while swallowing, speech alternation, or taste impairment observed during the out-patient department follow-up.

Laryngoscopic images were obtained in patients within 24 h after surgery (Figure 1). The larynx was sub-segmented to the vocal fold and interarytenoid region.

**Figure 3:**
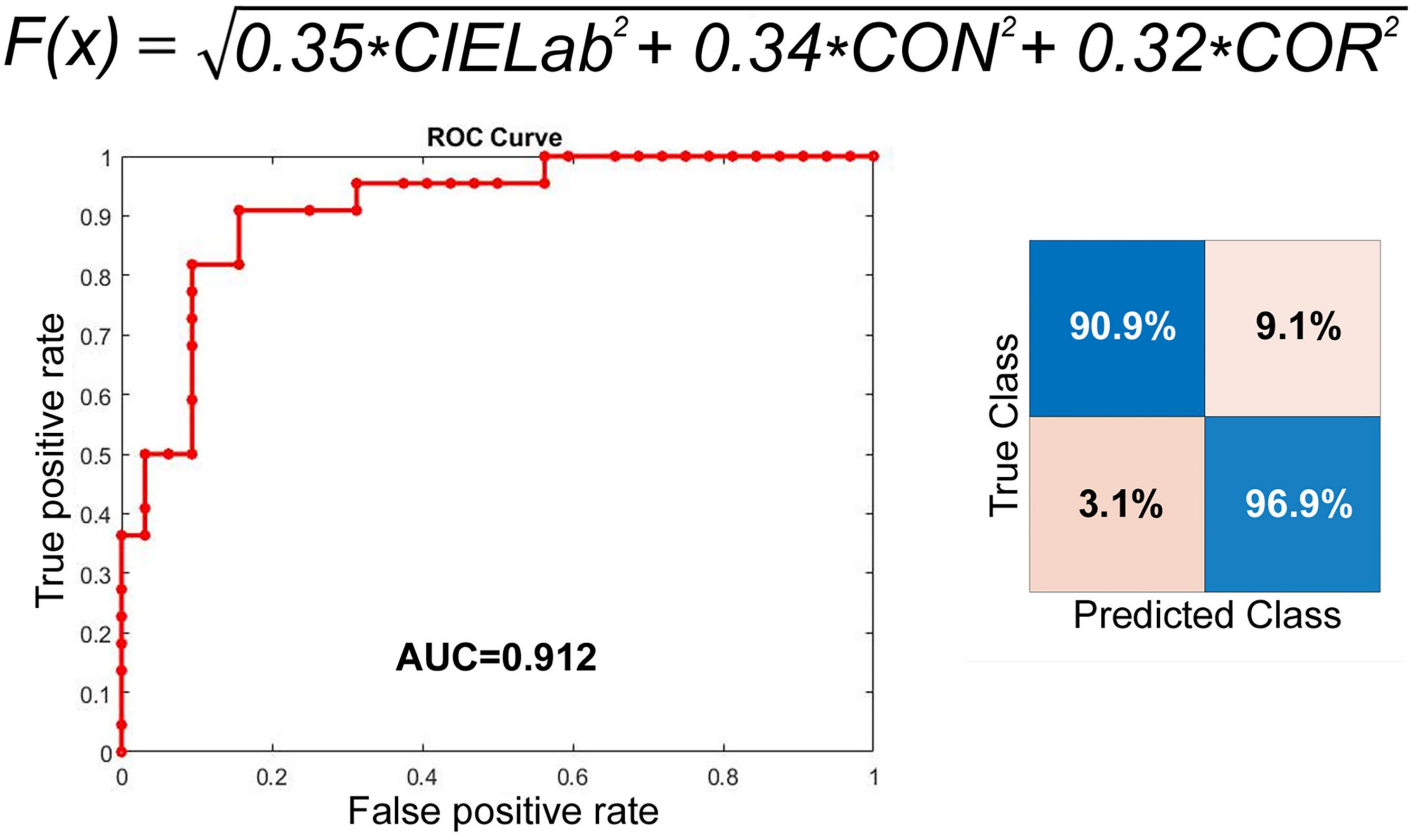
ROC curve of our function index to predict post-intubation larygneal inflammation. Our measurement has a sensitivity of 90.9% and a specificity of 96.9%.

To analyze the degree of erythema and inflammation after surgery, the texture profiles of these two regions, including contrast, energy, correlation, and homogeneity, under different hue conditions (R, G, B) were calculated, respectively. In the true fold region, the AUC of hue, contrast, and correlation in R color space were 0.679, 0.739, and 0.607, respectively. While in the interarytenoid region, significantly better discrimination was found and the AUC of hue, contrast, and correlation were 0.873, 0.829, and 0.812, respectively (Table 1). This might indicate that the interarytenoid region played a more important role in identifying the post-operative laryngeal inflammation, in terms of color or texture changes. As for other color space measurement, all eigenvalues showed a significant difference before and after surgery in the interarytenoid region, except for energy measure in G color. Spectroradiometric objective color measurements and the CIELab were used together with contrast and correlation profile from texture analysis. All measures showed a statistically significant increase after intubation (all *p* < 0.001). Receiver operating characteristic (ROC) curves were used to determine the cutoff values to detect patients with a high risk of post-operative laryngeal inflammation. We yielded an overall classification accuracy of 84.9% (by confusion matrix), with an area under the ROC curve (AUC) of 0.912. High area under the ROC curve (AUC) values suggested that our function index was “outstanding” at classifying a high risk for post-operative laryngeal inflammation (Figure 3). This indicates that our equipment and processing were not only accurate but also has a high measurement of discrimination.

**Table 1:** ROC-AUC curve analysis of different hue and texture features for vocal fold (VF) and interarytenoid region (IA).

**ROC-AUC**	**Hue**			**Contrast**			**Energy**			**Correlation**			**Homogeneity**		
	***R***	***G***	***B***	***R***	***G***	***B***	***R***	***G***	***B***	***R***	***G***	***B***	***R***	***G***	***B***
VF	0.679	0.61	0.56	0.739	0.685	0.665	0.68	0.675	0.68	0.603	0.58	0.5	0.71	0.695	0.61
IA	0.873	0.835	0.833	0.829	0.807	0.814	0.826	0.776	0.777	0.812	0.8	0.762	0.714	0.716	0.752

*The discrimination was significantly better in IA than in VF*.

**Figure 4:**
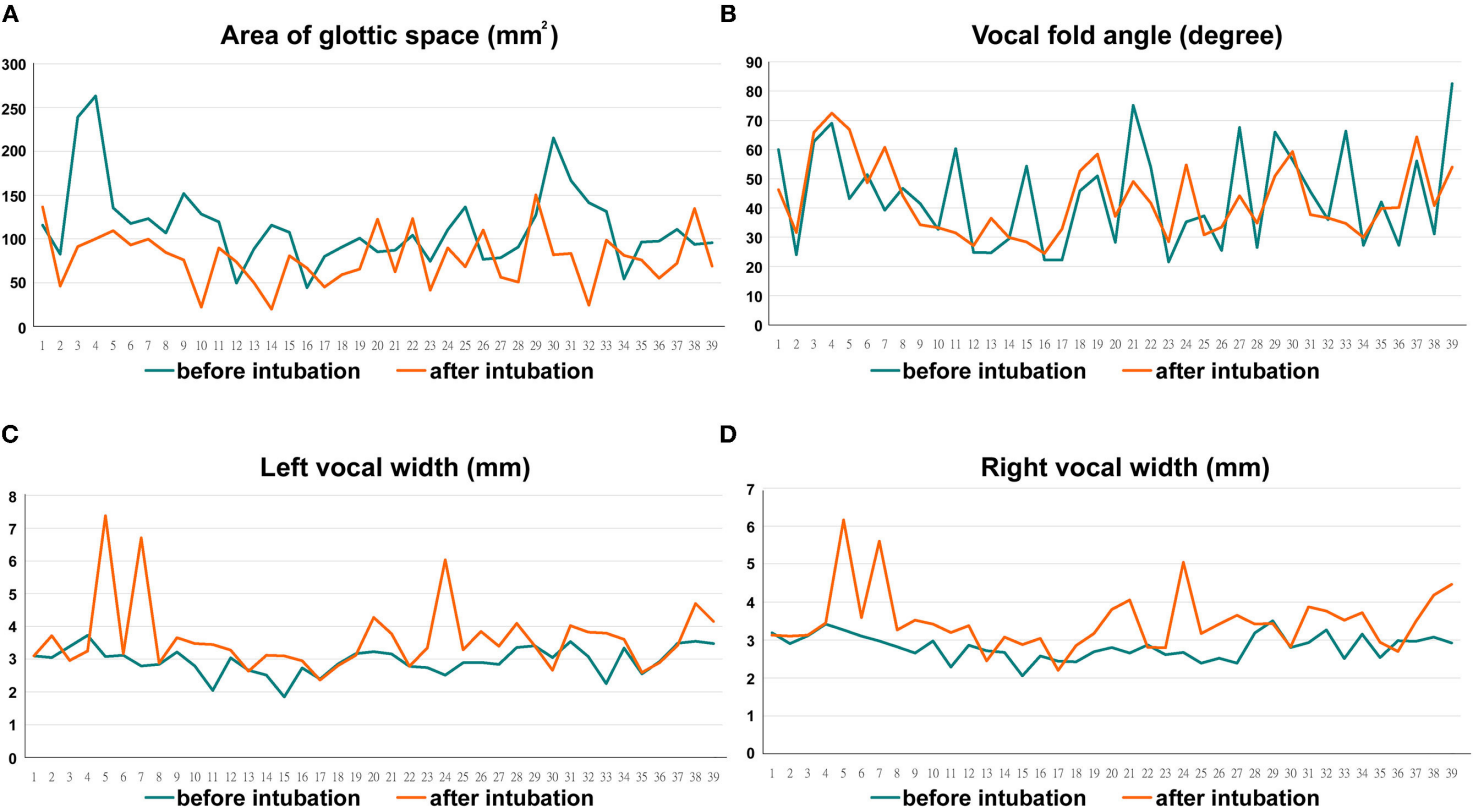
The changes in maximum value of the glottic area (31.2% decrease) **(A)**, vocal fold angle (2.58% decrease) **(B)**, and vocal fold width (Left vs. right: 22.01 vs. 23.46% increase, respectively) **(C&D)**.

The changes in maximum value of the glottic area, vocal fold angle, and vocal fold width (at midpoint) are summarized in Figure 4. The glottic area showed a significant decrease of 31.2% after operation. Similar trends toward smaller vocal angle with 2.58% decrease after extubation, but without statistical significance. Both the decreases in the glottic area and vocal fold angle indicated laryngeal edema that resulted in decreased airway patency. The vocal length revealed no significant change, but both the vocal width and area significantly increased after surgery. The width increased from 2.96 to 3.61 mm on left, while from 2.81 to 3.47 mm on the right. Although without statistical significance, the vocal width on the right increased a bit more than the one on the left (23.46 vs. 22.01%). Our result demonstrated that although color and texture change in the true fold region was obscure, the absolute value of the metric dimension change still has clinical benefit in identifying post-operative inflammatory changes.

The pre-operative and post-operative retropalatal depth and CSAOI were automatically identified and calculated, as shown in our previous work (6). With a specially designed angle laser line (Figure 2), the depth can be calculated by our module (θ = 8.5 degrees) and can provide the scaling reference to determine the CSAOI. We converted the data measured after the operation relative to the data measured before the surgery into percentages, to compare the post-operative changes. The post-operative retropalatal depth and CSAOI were significantly deeper and larger than the baseline, and the velopharyngeal spaces were successfully widened. Most of the patients' retropalatal depth (82%; *n* = 32) and CSAOI (100%; *n* = 39) showed significant increase, reaching their peaks in the 4th week after surgery (Table 2). After that, both measures declined slightly.

**Table 2:** Pre-operative and post-operative retropalatal depth (%) and CSAOI (%) measurement.

	**Mean** **±** **SD**				
	**Pre-**	**Post-W1**	**Post-W2**	**Post-W4**	**Post-12**
Retropalatal depth (%)	100	111.48 ± 6.95	135.75 ± 17.31	160.59 ± 34.21	151.95 ± 33.77
Cross-sectional area (%)	100	128.01 ± 11.32	148.46 ± 21.42	168.81 ± 31.89	159.25 ± 26.65

## Discussion

This study yielded several interesting results after statistical analysis of the association between endotracheal intubation and airway mucosal appearance, including color and texture profiles. The CIELab, or CIE L^*^ a^*^ b^*^, color system represents quantitative relationship of colors on three axes: L^*^ value indicates lightness, and a^*^ and b^*^ are chromaticity coordinates. CIELab is the reference color model used by the paper making and graphic arts industries. One advantage is that using CIELab instead of hue (RGB color space) allows for better control over differences in lighting. Texture analysis refers to the characterization of regions in an image by their texture content. It attempts to quantify intuitive qualities described by terms such as rough, smooth, silky, or bumpy as a function of the spatial variation in pixel intensities. In this sense, the roughness or bumpiness refers to variations in the intensity values, or gray levels (10). Texture analysis is useful in medical image processing, such as in lung cancer identification of CT images (11). Texture analysis can be used to find the texture boundaries, called texture segmentation, which is only qualitatively assessable by the human visual system to a limited degree (12). By calculating CIELab and texture configurations, our function index allowed objective identification of post-operative inflammation in the larynx, which are indistinguishable even to the naked eye. Our approach yielded an optimal classification accuracy of 84.9% (by confusion matrix). The corresponding ROC curve provided an area under the curve equal to 0.92, indicating outstanding sensitivity (90.9%) and specificity (96.9%). Our quantification allowed for objective visualization of the larynx by creating a quantified color and texture profile, independent of subjective clinical observations. Subsequently, we compared the hue and texture profiles over the interarytenoid region before and after endotracheal extubation. Regardless of the three R,G and B color spaces, all measures (energy, correlation, homogeneity, and contrast) showed significant distinction before and after the operation, with AUC of 0.714–0.87 (Table 1). Among them, the discriminative power of R channel was the best. As for the true vocal fold region, the analysis showed insufficiency to no discrimination, with AUC around 0.5~0.74. The better discrimination of R channel was consistent with the clinical setting of inflammatory responses on mucosal surfaces. The result suggested the inflammation over the interarytenoid region was more severe than the true vocal cord region. The reason may be that the endotracheal tube was placed mainly upon the interarytenoid mucosa. Our results indicated that the described technique are both accurate and applicable, which would allow a clinician to approach post-operative laryngeal inflammation easily and less invasively. Hence, it will benefit the clinician who would otherwise make a differential diagnosis based solely on subjective interpretation of non-specific laryngeal signs.

Trauma during endotracheal intubation could cause post-operative laryngeal complications with vocal cords edema or hematoma (13). Inflammation changes the tissue composition leading to higher water content and edema leads to significant changes in laryngeal mucosal thickness, including decreases the glottic area and vocal angle and increases the vocal fold width and area. For the first time, our research has demonstrated subtle changes in larynx that were difficult to objectively quantify in the past. Vocal fold mass is a parameter of great significance to the pitch range and timbre of a voice, while the increased vocal width can lead to post-operative dysphonia or pitch break. In the past, *in vivo* detection of metric dimensions of laryngeal structure was very difficult due to lack of suitable tools. Several attempts have been made by previous researches, including estimation through radiography or from cadavers (14, 15). But none of those could directly reflect the physiological vocal fold condition in real time and the inaccurate measurement made precise interpretation of the data difficult. In the present study, the described technique enabled the detection of absolute spatial laryngeal dimension in living subjects, and the measurement was both accurate and physiological. Vocal fold width increased more on the right than on the left. We had once discussed this with anesthesiologists and they supposed it was because they usually fixed the tracheal intubation on the right side. However, in this series, the tubes were all fixed in the midline area of the mouth during surgery and the tube position should not affect our result. Therefore, we hypothesized that the process of intubation should be responsible for this phenomenon. Since most anesthesiologists were right-handed, there was reason to assume the right vocal cord sustains more stress and impact during intubation. Although there was no statistical significance, the wider and heavier right vocal fold might hint that the intubation process played an important role in the formation of post-operative laryngeal edema and we should improve the insertion process to avoid sequelae.

Unlike other studies that used the subjective measures with the Epworth Sleepiness Scale as the evaluation for post-operative changes in throat (16), for the first time we directly determined and calculated the anatomic change after surgery with 12 weeks follow-up. The significant increase in the post-operative retropalatal depth and CSAOI provided a substantial evidence that palatoplasty was actually effective to enlarge oropharyngeal dimension. Palatoplasty was supposed to widen the lateral oropharyngeal airway, but it was difficult to prove it quantitatively in the past, especially in an outpatient setting. At the same time, it was also controversial whether palatoplasty can result in the elevation of the soft palate. In current study with quantitative measurement, we have provided evidence that palatoplasty can preserve the lateral pharyngeal wall tension and enlarge the retropharyngeal space. Most of the measures reached their maximums in the 4th week after the surgery, and slightly decreased in the 12th week (Table 2). We supposed that there was improved post-operative tissue swelling in the first 4 weeks, combined with tension in the oropharyngeal wound caused by collagen fibers in the remodeling phase during wound healing, which would be eliminated ~3–4 weeks after the oropharyngeal wound healed. There was scar remodeling afterward, while the continuous reorganization of collagen fibers in the soft palate led to granulation tissue regression and CSAOI increase, but the absorption and loss of suture tension might decrease the CSAOI and retropalatal depth. According to the manufacturing information, the *in-vivo* tensile strength retention of 3-0 Polysorb™ braided absorbable suture is 3 weeks and its absorption profile is around 70 days. This was in line with our finding that both the CSAOI and the retropalatal depth decreased after they reached their plateau. The scar remodeling process usually peaks around 3–6 months and lasts for up to a year. This may be one of the reasons for the recurrence of oropharyngeal stricture after surgery. In this context, early detection and close monitoring through a convenient, non-invasive examination that is less time-consuming and more patient-compliant are essential.

In most previous studies, the degree of upper airway obstruction of patients with obstructive sleep apnea was evaluated with polysomnography, multiple sleep latency tests, Muller maneuver, etc. drug-induced sleep endoscopy is widely used now, due to its superiority to dynamic observation of volumetric changes in the upper airway (17). However, drug-induced sleep endoscopy is more invasive and depends on the clinicians' subjective judgment. The real size of the oropharynx, including the retropalatal depth and CSAOI, was not quantitatively analyzed in the past, until a recent study introduced endoscopic morphometric measurement with a laser module (6). This oropharyngeal laryngoscopy can easily and automatically measure the retropalatal depth and CSAOI with the laser module (Figure 2). Also, this examination can be performed at outpatient departments—as well as patients' post-operative evaluation—so the degree of narrowing of patients' oropharyngeal structure can be quickly assessed. The practicality of this device does not only bring convenience to the clinicians, but also increases the patients' willingness to undergo repeated follow-up examinations.

Despite considerable effort and care during operation, post-operative airway complication will still occur. Earlier studies have not shown any significant differences in the experience of anesthesia personnel and the incidence of post-operative sequelae (18). Previous researches have demonstrated inconclusive association between post-operative sequelae and insertion technique, or care during anesthesia (19). The lack of the optimal design trial with appropriate objective quantitative evaluation tools might be the main reason. As a result, it has been difficult to devise protocols to minimize such injuries. With the described technique, we can conduct a prospective study to compare different insertion techniques among different surgery, variable anesthetic factors, or different intubating conditions. A major limitation of current study is the small population and lack of a control group. We did not include other factors such as the experience of the intubator, extubation bucking, or the use of a suction catheter. We did not control or standardize the anesthesia protocol, but no persons in training were allowed to intubate in this series. The strength of this study design is that the results reflect a real-life situation. The performance of our predictive models is also fairly good. Hence, our tool may be important for early assessment at out-patient departments, as many patients do not require other complicated examinations at first.

## Conclusion

Our endoscopic laser module was the first truly objective and quantitative measurement of laryngeal and oropharyngeal structures before and after endotracheal Intubation for palatoplasty. The increased contrast texture and the trend to red were compatible with post-operative inflammatory response in the interaryteonid region. Furthermore, increased vocal fold mass and reduced glottic space suggested post-operative vocal fold edema, with higher water content and increased mucosal thickness. We also provided direct evidence that palatoplasty can effectively enlarge the retropharyngeal space by creating anchorage points to suspend the lateral pharyngeal wall and the soft palate. Here additional information collected by different imaging modalities would help to solve multiple current unmet needs in post-operative airway inflammation.

## Data Availability Statement

The raw data supporting the conclusions of this article will be made available by the authors, without undue reservation.

## Ethics Statement

The Research Protocol (No: 1-108-05-132) has been reviewed and approved by the Institutional Review Board of Tri-Service General Hospital. The patients/participants provided their written informed consent to participate in this study.

## Author Contributions

P-RL: acquisition of data, data analysis and interpretation, and article and images review/editing. CK: study design and critical article review/editing. S-CL: study design, data collection, literature search, images editing, article drafting, and article submission. All authors contributed to the article and approved the submitted version.

## Funding

This research was partially supported by Tri-Service General Hospital, National Defense Medical Center (MND-MAB-D-111116).

## Conflict of Interest

The authors declare that the research was conducted in the absence of any commercial or financial relationships that could be construed as a potential conflict of interest.

## Publisher's Note

All claims expressed in this article are solely those of the authors and do not necessarily represent those of their affiliated organizations, or those of the publisher, the editors and the reviewers. Any product that may be evaluated in this article, or claim that may be made by its manufacturer, is not guaranteed or endorsed by the publisher.
